# DUSP16 is an epigenetically regulated determinant of JNK signalling in Burkitt's lymphoma

**DOI:** 10.1038/sj.bjc.6605711

**Published:** 2010-06-15

**Authors:** S Lee, N Syed, J Taylor, P Smith, B Griffin, M Baens, M Bai, K Bourantas, J Stebbing, K Naresh, M Nelson, M Tuthill, M Bower, E Hatzimichael, T Crook

**Affiliations:** 1 Laboratory of Cancer Genetics and Epigenetics, Breakthrough Breast Cancer, Institute of Cancer Research, Fulham Road, London, UK; 2 Department of Virology, Imperial College School of Medicine, St Mary's Campus, Norfolk Place, London, UK; 3 Human Genome Laboratory, Department of Human Genetics, Flanders Interuniversity Institute for Biotechnology (VIB), Katholieke Universiteit Leuven, Herestraat 49, B-3000 Leuven, Belgium; 4 Department of Pathology, University Hospital of Ioannina, St Niarchou Avenue, Ioannina, Greece; 5 Department of Hematology, University Hospital of Ioannina, St Niarchou Avenue, Ioannina, Greece; 6 Department of Medical Oncology, Imperial College London, Charing Cross Hospital, London, UK; 7 Department of Pathology, Hammersmith Hospital, London W9, UK; 8 Department of HIV Medicine, Imperial College London, Chelsea and Westminster Hospital, Fulham Road, London, UK; 9 Department of Oncology, Imperial College London, Chelsea and Westminster Hospital, Fulham Road, London, UK

**Keywords:** DUSP, Epigenetics, HIV, Burkitt's lymphoma

## Abstract

**Background:**

The mitogen-activated protein kinase (MAPK) phosphatases or dual specificity phosphatases (DUSPs) are a family of proteins that catalyse the inactivation of MAPK in eukaryotic cells. Little is known of the expression, regulation or function of the DUSPs in human neoplasia.

**Methods:**

We used RT–PCR and quantitative PCR (qPCR) to examine the expression of *DUSP16* mRNA. The methylation in the DUSP16 CpG island was analysed using bisulphite sequencing and methylation-specific PCR. The activation of MAPK was determined using western blotting with phospho-specific antibodies for extra-cellular signal-related kinase (ERK), p38 and c-Jun N-terminal kinase (JNK). The proliferation of cell lines was assessed using the CellTiter 96 Aqueous One assay.

**Results:**

The expression of DUSP16, which inactivates MAPK, is subject to methylation-dependent transcriptional silencing in Burkitt's Lymphoma (BL) cell lines and in primary BL. The silencing is associated with aberrant methylation in the CpG island in the 5′ regulatory sequences of the gene blocking its constitutive expression. In contrast to BL, the CpG island of DUSP16 is unmethylated in other non-Hodgkin's lymphomas (NHLs) and epithelial malignancies. In BL cell lines, neither constitutive nor inducible ERK or p38 activity varied significantly with DUSP16 status. However, activation of JNK was increased in lines with DUSP16 methylation. Furthermore, methylation in the DUSP16 CpG island blocked transcriptional induction of DUSP16, thereby abrogating a normal physiological negative feedback loop that limits JNK activity, and conferred increased cellular sensitivity to agents, such as sorbitol and anthracycline chemotherapeutic agents that activate JNK.

**Conclusion:**

DUSP16 is a new epigenetically regulated determinant of JNK activation in BL.

Burkitt's Lymphoma (BL) is a highly aggressive B cell tumour often presenting in extra-nodal sites or as acute leukemia (Blum *et al*, 2004). In the World Health Organization (WHO) classification, three clinical variants of the disease are described. Endemic BL, the most chemosensitive subtype, affects children and young adults mainly in Africa and has been strongly correlated with Epstein–Barr virus (EBV) infection. In contrast, sporadic BL occurs worldwide and is predominantly EBV negative (Brady *et al*, 2008). Immunodeficiency-associated BL occurs as a well-recognised clinical feature of acquired immune deficiency syndrome. The molecular hallmark of BL is the activation of the *c-myc* oncogene through reciprocal chromosomal translocations that juxtapose c-myc on chromosome 8 to the immunoglobulin (Ig) heavy chain locus on chromosome 14 (80% of cases) or the *κ*- or *λ*-light chain locus on chromosome 2 (10% of cases; Aldoss *et al*, 2008).

The mitogen-activated protein kinases (MAPKs) function in co-ordinating cellular processes, such as proliferation and apoptosis. There are at least three major groups of MAPK recognised. These comprise the extra-cellular signal-related kinases (ERKs), Jun N-terminal kinase (JNK) and a p38 MAPK (Boutros *et al*, 2008). The MAPK phosphatases or dual specificity phosphatases (DUSPs) are a family of proteins that catalyse the inactivation of MAPK in eukaryotic cells and are so-called because of their ability to remove phosphate groups from both threonine and tyrosine residues in MAPK (Theodosiou and Ashworth, 2002). According to sequence homology, substrate selectivity and sub-cellular localisation, the DUSPs can be classified into distinct groups. Group I comprises the nuclear, inducible DUSPs; DUSP1, DUSP2, DUSP4 and DUSP5 that target the primary MAPK, ERK, JNK and p38 MAPK. Group II consists of cytoplasmic DUSPs that predominantly target ERK and includes DUSP6, DUSP7 and DUSP9. On account of its unique N-terminal structure, DUSP 10 is placed into group III. It is both nuclear and cytoplasmic and targets p38 MAPK and JNK. Group IV contains DUSP8 and DUSP16 that possess unique C-terminal sequences. In comparison to the other DUSP groups, relatively little is known about the group IV DUSPs (Keyse, 2008). However, DUSP16 is reported to inhibit MAPK activity in the order JNK >> p38 > ERK, with a preferential inhibition of JNK (Tanoue *et al*, 2001). DUSP16 dynamically interacts with the JNK3 scaffold protein *β*-arrestin 2 (Willoughby and Collins, 2005). The ability of DUSPs to negatively regulate MAPK implies that they may function as tumour suppressors (Theodosiou and Ashworth, 2002). DUSP16 may suppress the transformed phenotype of rodent fibroblasts expressing bcr-abl, consistent with a tumour suppressor function, although definitive evidence in support of this hypothesis is lacking (Hoornaert *et al*, 2003).

There is now good evidence that epigenetic inactivation, frequently through methylation-dependent transcriptional silencing, is a common mechanism of inactivation of genes in cancer (Stebbing *et al*, 2006; Esteller, 2007). However, only a small number of transcriptionally silenced genes, such as *p73* and fragile histidine triad (*FHIT*), have been described in BL (Corn *et al*, 1999; Lindstrom and Wiman, 2002; Hussain *et al*, 2004; Syed *et al*, 2006).

We have previously reported the use of subtraction PCR to identify genes downregulated in B lymphomas and from these studies observed methylation-dependent transcriptional silencing of the polo-like kinase Snk/Plk2 (Syed *et al*, 2006), which showed a strong, but not absolute, selectivity among B cell neoplasias, for BL. In this study, we report the methylation-dependent transcriptional silencing of a second gene, *DUSP16*, which demonstrates a remarkable selectivity for BL.

## Materials and methods

### Cell lines and tissues

Burkitt's lymphoma and EBV-immortalised B lymphoblastoid cell lines (LCLs) were maintained in Roswell Park Memorial Institute 1640 (Gibco Invitrogen, Carlsbad, CA, USA) medium containing 10% foetal bovine serum. The following cell lines were used in our study: nine BL cell lines (AW Ramos, BL2, BL41, Dante, DG75, Mak-1, Mutu, Rael and Ramos); one mantle-cell lymphoma (MCL) cell line (JUM2); one transformed follicular lymphoma (FL) cell line (DOHH2); one diffuse large B-cell lymphoma (DLBCL) cell line (LK6); and 10 LCLs (CR, IB4, JAC, JMV25, Otis, PD, GM12794, GM13021, GM13022 and GM16756). The following carcinoma cell lines were analysed for *DUSP16* CpG island methylation: nine epithelial ovarian cancer cell lines (1847, TR175, SKOV3, OVCAR3, OVCAR433, OVCAR5, OVCAR8, JAMA2 and A2780) and 15 breast cancer cell lines (MCF102, MDA MB157, MDA MB231, MDA MB361, MDA MB436, MDA MB 453, MDA MB 468, MCF7, NCI, GI101, T47D, BT20, CAL51, ZR75 and BT474).

For the activation of the JNK MAPK pathway, the following treatments were used: sorbitol (Sigma-Aldrich Ltd., Dorset, UK) at 0.4 M for 30 min; and cisplatin (Faulding Pharmaceuticals) at 50 *μ*g ml^−1^ from 5 min to up to 8 h. When titrating the concentrations of cisplatin, 1, 10, 20, 30 and 50 *μ*g ml^−1^ were tested at 6 h after the addition. For the stimulation of the ERK and p38 MAPK pathway, the following treatments were used: 40% foetal calf serum (Invitrogen, Paisley, UK) for 30 min; epidermal growth factor at 0.1 *μ*g ml^−1^ for 10 min; phorbol 12-myristate 13-acetate (Sigma-Aldrich Ltd.) dissolved in dimethyl sulphoxide at 10 ng ml^−1^ for 10 min. For the stimulation of the p38 MAPK pathway, anisomycin (Sigma-Aldrich Ltd.) at 10 *μ*g ml^−1^ for 30 min was used.

Tissues were collected after local ethics committee's approval. Primary lymphomas were obtained as formalin-fixed, paraffin-embedded biopsies. The diagnosis of malignant lymphoma was made by morphological and immunohistochemical analysis according to the WHO classification. Two expert haematopathologists (KN and MB) reviewed the diagnosis of the NHL cases and the sporadic and human immunodeficiency virus (HIV)-associated BL. The histological types for the non-Hodgkin's lymphoma (NHL) samples were: diffuse large cell lymphoma (DLBCL, *n*=10), follicular lymphoma (FL, *n*=10), mantle cell lymphoma (MCL, *n*=10), marginal zone lymphoma (MZL, *n*=10) and BL: endemic (*n*=45), sporadic (*n*=8) and HIV-associated (*n*=4).

Genomic DNA was obtained using the DNeasy Mini kit (Qiagen, Crawley, UK) or by proteinase K/phenol method.

### Generation of cell lines ectopically expressing DUSP16

The DUSP16 expression plasmid, MSCVpuroDUSP16, used to generate cell lines stably overexpressing DUSP16 has been described previously (Hoornaert *et al*, 2003). One of the DG75 DUSP16 overexpressing lines was generated by electroporation. For this, DG75 BL cells (10 × 10^6^) were re-suspended in 800 *μ*l Roswell Park Memorial Institute 1640 medium mixed with 20 *μ*g MSCVpuroDUSP16 vector. Cells were electroporated at 975 mFA and 265 V in a 4-mm cuvette, placed on ice for 1 min and added to 10-cm^2^ dish containing 9 ml of pre-warmed Roswell Park Memorial Institute 1640 medium. Puromycin (Sigma-Aldrich Ltd.) selection 0.5 *μ*g ml^−1^ was added to the cells at 2 days after electroporation. Fresh medium and drugs were added every 2–3 days until distinct colonies were formed. Other DG75 DUSP16 overexpressing cell lines were generated again by electroporation, but using the cell line nucleofector kit V from Amaxa (Amaxa Biosystems, Koeln, Germany). A total of 2 *μ*g of MSCVpuroDUSP16 was transfected into 2 × 10^6^ DG75 cells using solution V and programme A-24. Cells were selected in 0.3 *μ*g ml^−1^ puromycin 1 day after electroporation.

### Cell proliferation assay

Cell proliferation assays were carried out using the CellTiter 96 Aqueous One solution assay (Promega, Southampton, UK) according to the manufacturer's instructions, except that 10 *μ*l of Aqueous One solution was used instead of 20 *μ*l and cells were plated in 200 *μ*l of culture medium instead of 100 *μ*l. Parental DG75 cells and DUSP16 overexpressing clones were plated in each well of a 96-well plate. Cells were then either left untreated or exposed to serial dilutions of sorbitol (0.025–0.8 M), cisplatin (3.125–100 *μ*g ml^−1^) and doxorubicin (1.25–40 *μ*g ml^−1^) and analysed at various times after addition of the drug. Each treatment was performed in quadruplicate for each cell line.

### Bisulphite modification and methylation-specific PCR

Genomic DNA was extracted from cell pellets using the DNeasy Mini Kit (Qiagen) according to the manufacturer's instructions. The DNA (0.5 *μ*g) was modified by sodium bisulphite using the EZ DNA Methylation Kit (Zymo Research, Orange, CA, USA). This process converts unmethylated cytosine residues to uracil, whereas methylated cytosine residues remain unchanged. Methylation-specific PCR was then carried out to determine the methylation status of DUSP16. Bisulphite-modified DNA was used as a template for PCR with primers specific for methylated or unmethylated alleles. CpGenome Universal Methylated DNA (Chemicon Europe, Chandlers Ford, Hampshire, UK) and normal human unmethylated DNA were used as positive and negative controls, respectively, in each experiment.

The primer sequences were as follows:

For DUSP16:

5′-CCACCCTTTCAAAAAACAACATAAAAACA-3′ (unmethylated forward),

5′-AGTGTATTTATTGTGATTTTGTGTTTGGTT-3′ (unmethylated reverse),

5′-ACCCTTTCGAAAAACGACGTAAAAACG-3′ (methylated forward) and

5′-GTATTTATTGCGATTTCGCGTTCGGTC-3′(methylated reverse).

The PCR conditions were as follows: eight cycles of 95°C for 2 min, 60°C for 30 s and 72°C for 30 s were followed by 32 cycles of 95°C for 30 s, 60°C for 30 s and 72°C for 30 s and then a final extension was carried out at 72°C for 5 min. The PCR products were resolved by electrophoresis using 2% agarose gels, stained with ethidium bromide, and visualised using a transilluminator.

### Bisulphite sequencing

The bisulphite-modified genomic DNA (see above) was used as a template in PCR. Two sets of primers were used to sequence the DUSP16 CpG. The primer sequences were as follows:

For DUSP16: 5′-TTATTTTTTAGAGAAGGAGAAGATAATATA-3′ (forward 1) and 5′-CCAAAACCACTTACTTTTAAAACC-3′ (reverse 1),

5′-TTTTTTGAGGGAATTGGGAG-3′ (forward 2) and

5′-TAATAAAAACAATCAAACCAAAACC-3′ (reverse 2).

Reaction conditions for PCR were as follows: an initial incubation at 95°C for 5 min was followed by 28 cycles of 95°C for 30 s, 55°C for 30 s and 72°C for 30 s and a final extension was performed at 72°C for 5 min. The PCR products were purified using a PCR purification kit (Qiagen), ligated into a TA-cloning vector (Invitrogen), and transformed into top 10 *Escherichia coli*-competent cells (Invitrogen). Colonies were grown on Luria–Bertani agar plates under ampicillin and blue/white selection. Plasmid DNA was used for sequencing with the BigDye Terminator Cycle kit (PE Applied Biosystems, Warrington, UK) and reverse primers. For each sample a minimum of six clones were sequenced.

### Methylation reversal

Cells were treated with 5 *μ*M 5′azacytidine (5′AZA; Sigma-Aldrich Ltd.) for 5 days followed by a combination of 5′AZA and 300 nM Trichostatin A (Sigma-Aldrich Ltd.) for a further 1–2 days. The cells were split every 2–3 days with the addition of fresh drug. After drug treatment, cells were collected for RT–PCR.

### Analysis of gene expression

Total RNA was extracted using Trizol (Invitrogen). The complementary DNA was synthesized from 1 *μ*g total RNA using oligo (dT) primers and the ImProm-II Reverse Transcriptase Kit (Promega). The expression of DUSP16 was analysed by RT–PCR and real-time PCR (quantitative PCR). Primer sequences were as follows:

For DUSP16: 5′-GCACACCACCATTACATCATCG-3′ (forward) and

5′- AACAGTCTGAAGAGAGAGAGGC-3′ (reverse)

and the product size was 373 bp. The PCR conditions were as follows: an initial denaturation at 95°C for 5 min was followed by 28 cycles of 95°C for 30 s, 55°C for 30 s and 72°C for 30 s and a final extension was carried out at 72°C for 5 min. Reactions were resolved on 2% agarose gels and visualised on a transilluminator after staining with ethidium bromide. The glyceraldehyde 3-phosphate dehydrogenase (*GAPDH*) was co-amplified as a control gene. The primer sequence for *GAPDH* is

5′-TGAAGGTCGGAGTCAACGGATTT-3′ (forward) and

5′-GCCATGGAATTTGCCATGGGTGG-3′ (reverse).

The PCR conditions were as follows: an initial denaturation at 95°C for 5 min was followed by 23 cycles of 95°C for 1 min, 59°C for 55 s and 72°C for 45 s and a final extension was performed at 72°C for 7 min.

Quantitative RT–PCR was performed in an ABI PRISM 7700 Sequence Detection System (PE Applied Biosystems, Weiterstadt, Germany) using sequence-specific probes for DUSP16 and GAPDH (PE Applied Biosystems). The TaqMan universal PCR master mix (PE Applied Biosystems) was used and each sample was analysed in triplicate.

### Proteasome inhibition

The BL and LCL cells were either untreated or serum-starved overnight and then treated with cisplatin (50 *μ*g ml^1^) for 6 h alone or with 30 *μ*M of a proteasomal inhibitor (MG132; Sigma-Aldrich Ltd.) for either 3 or 6 h.

### Western blotting

To obtain protein from untreated exponentially growing cells, cells were collected and lysed in lysis buffer (50 mM Tris–HCl (pH 7.5), 250 mM NaCl, 0.1% NP-40, 5 mM EDTA, 50 mM NaF, 1 mM phenylmethylsulphonyl fluoride with protease inhibitor cocktail; Roche, Indianapolis, IN, USA). Cleared lysates were assayed for protein concentration by using the Bio-Rad (Hemel Hempstead, Hertis, UK) protein assay system and subjected to immunoblotting. Bound primary antibodies were detected with either horseradish peroxidise-conjugated goat antirabbit antibody or horseradish peroxidise-conjugated goat antimouse antibody (Dako UK Ltd, Cambridgeshire, UK). Enhanced chemiluminescence western blotting detection reagents were purchased from Amersham (Freiberg, Germany). The primary antibodies purchased from Cell Signaling Technologies were used: anti-SAPK (stress-activated protein kinase)/JNK MAPK, anti-p44/42 MAPK, anti-p38 MAPK, anti-phospho-SAPK/JNK (Thr183/Tyr185), anti-phospho-p44/42 MAPK (Thr202/Tyr204) and anti-phospho-p38 MAPK (Thr180/Tyr182). Anti-CtBP (C-terminal Binding Protein) was a kind gift from Qinghong Zhang and has been described previously (Wang *et al*, 2006). The mouse monoclonal antibody PC10 was used for detection of proliferating-cell nuclear antigen (1 : 10 000).

## Results

### Transcriptional silencing of DUSP16 in Burkitt's Lymphoma

**Figure 1:**
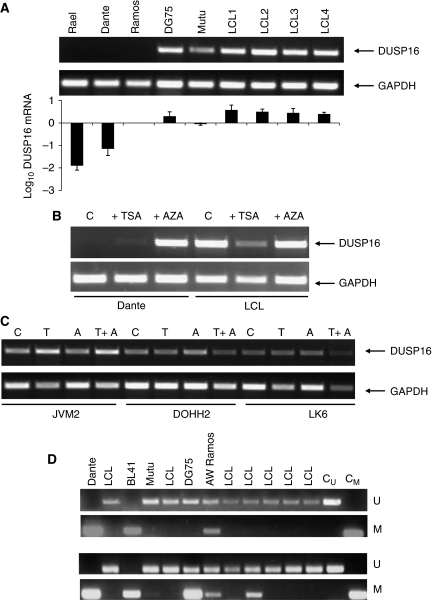
Methylation-dependent silencing of DUSP16 expression in BL. (**A**) RT–PCR and qPCR analysis of *DUSP16* mRNA expression in BL cell lines and LCLs. Total RNA was prepared from exponentially growing cells as described in Materials and Methods section, and expression of *DUSP16* and control gene *GAPDH* mRNA analysed by RT–PCR (upper panel), and DUSP16 by qPCR (lower panel) as described in Materials and Methods section. In the qPCR analysis, expression (±1 s.d.) is relative to Ramos. (**B**) The expression of DUSP16 is reactivated by demethylation in BL but is unaffected in LCL. Exponentially growing cells were treated with 5′azacytidine (5′AZA) or Trichostatin A (TSA) as indicated. cDNA was prepared as described in Materials and Methods section and expression of *DUSP16* and the control gene *GAPDH* determined. C indicates control cells untreated with either 5′AZA or TSA. (**C**) *DUSP16* mRNA is expressed and not epigenetically regulated in non-BL NHL. Expression of *DUSP16* and *GAPDH* mRNA was analysed by RT–PCR in JVM2, DOHH2 and LK6 and cell lines with or without exposure to 5′AZA, TSA and 5′AZA and TSA together as indicated. (**D**) The DUSP16 CpG island is methylated in BL cell lines, but not LCLs. MSP was performed as described in Materials and Methods section, using independent primer pairs in upper and lower panels. The figure shows unmethylated (U) and methylated (M) MSP reactions for each DNA sample, with control unmethylated (C_U_) and methylated (C_M_) reactions as shown.

We examined expression of the type IV DUSP, DUSP16, in BL cell lines using RT–PCR and quantitative PCR. Figure 1A shows RT–PCR and quantitative PCR analysis of levels of *DUSP16* mRNA (relative to Ramos in the quantitative PCR analysis). DUSP16 was expressed in all EBV-immortalised LCLs we studied, in each case at a higher level than in each of the BL cell lines analysed. The *DUSP16* mRNA was absent in the BL cell line, Rael, and virtually undetectable in Dante and at higher levels in DG75 and Mutu cell lines (Figure 1A).

**Figure 2:**
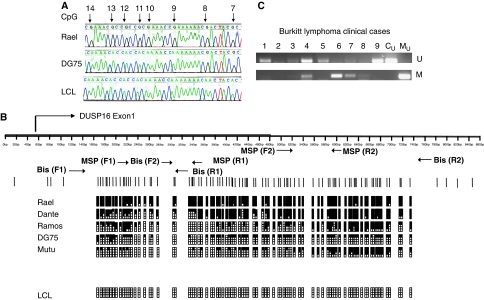
Methylation in the DUSP16 CpG island in BL cell lines and primary BL, but not in LCLs. (**A**) Sample bisulphite sequencing trace, showing complete methylation in Rael, partial methylation in DG75 and absence of methylation in the LCL. The arrows denote individual CpG dinucleotides numbered from the start of the CpG island. (**B**) Summary of bisulphite sequencing analysis of DUSP16 CpG island in BL and LCL. The figure shows a diagrammatic representation of the DUSP16 CpG island. CpG sites are shown as vertical lines. Methylated CpG dinucleotides are shown as black blocks, unmethylated CpGs as open blocks. Five levels of methylation are indicated: 0=no black blocks; 1–25%=one black block; 25–50%=two black blocks; 50–75%=three black blocks; 75–100%=four black blocks. Positions of the MSP and bisulphite sequencing primers are indicated. The CpG island is almost completely methylated in Rael, Dante and Ramos, partially methylated in DG75 and Mutu and unmethylated in the LCLs, consistent with expression of *DUSP16* mRNA in each cell line. (**C**) The DUSP16 CpG island is methylated in primary endemic BL. MSP was performed as described in Materials and Methods section. The figure shows unmethylated (U) and methylated (M) MSP reactions for each DNA sample, with control unmethylated (C_U_) and methylated (C_M_) reactions as shown.

There is a CpG island in the 5′ sequence of the *DUSP16* gene (http://www.genome.ucsc.edu/cgi-bin/hgGateway), raising the possibility that epigenetic silencing might underlie absent expression of the gene. To address this possibility, we tested whether expression of DUSP16 could be reactivated by demethylation. Cells were treated with 5′AZA or the histone deacetylase inhibitor, Trichostatin A, and expression was analysed by RT–PCR. In BL cell lines lacking *DUSP16* mRNA under the limiting PCR conditions used, expression was increased by the addition of 5′AZA. A representative experiment with the Dante BL cell line is shown in Figure 1B. In contrast, there was no change in *DUSP16* mRNA expression in LCLs after the treatment with 5′AZA. We also analysed the expression and the effect of demethylation in three additional NHL cell lines: JVM2 (MCL), DOHH2 (transformed FL) and LK6 (DLBCL). In each cell line, expression of DUSP16 was readily detectable in normally proliferating cells and did not change after 5′AZA and/or Trichostatin A treatment (Figure 1C). The presence of a CpG island and reactivation of expression in BL cell lines by 5′AZA treatment is consistent with methylation-dependent transcriptional silencing. Furthermore, the absence of increased expression after 5′AZA in non-BL NHL cell lines implies that silencing is specific for BL. To investigate this, we performed bisulphite sequencing of the entire sequence of the CpG island in several BL cell lines and in LCLs. These studies revealed the presence of dense CpG methylation in each of the BL cell lines that lacked *DUSP16* mRNA. In contrast, there was no methylation in the LCL that expresses DUSP16 (Figures 2A and B). In DG75 and Mutu, which express an intermediate level of *DUSP16* mRNA, there was partial methylation in the CpG island. We designed primers for methylation-specific PCR based on the sites of methylation revealed by bisulphite sequencing (Figure 2B) and initially tested a panel of BL cell lines and LCLs (Figure 1D). By methylation-specific PCR, the majority of BL cell lines were methylated with both primer sets. Dante and BL41 showed complete methylation (as evidenced by the absence of amplification with the U primer pair. Mutu, DG75 and AW Ramos were partially methylated, consistent with bisulphite sequencing data. Of the tested cell lines, only BL2 was unmethylated. Taken together, these results reveal a close correlation between low or undetectable expression of DUSP16 and methylation in the CpG island.

### DUSP16 methylation is specific for BL

These observations prompted us to analyse the methylation of DUSP16 in primary B cell neoplasms. We analysed a series of B lymphomas, including DLBCL, FL, MCL, marginal zone lymphoma and BL of endemic (*n*=45), sporadic (*n*=8) and human immunodeficiency virus-associated (*n*=4) sub-types (Table 1). Consistent with results obtained in cell lines, there was aberrant CpG methylation of DUSP16 in more than 50% (27 out of 45 cases) of the primary endemic BL cases analysed (Figure 2C). The methylation was also detected in two out of eight sporadic cases and in two out of four human immunodeficiency virus-associated cases, but there was no methylation in any other NHL types (Table 1). We next examined methylation in solid tumours. Methylation-specific PCR analysis of human breast and ovarian carcinoma cell lines and malignant melanoma cell lines revealed no detectable methylation in the DUSP16 CpG island, despite reproducible methylation in BL cell lines (data not shown). Together, these results reveal that methylation-dependent silencing of DUSP16 shows specificity for BL.

### DUSP16 methylation selectively deregulates stress-induced JNK activation

**Figure 3:**
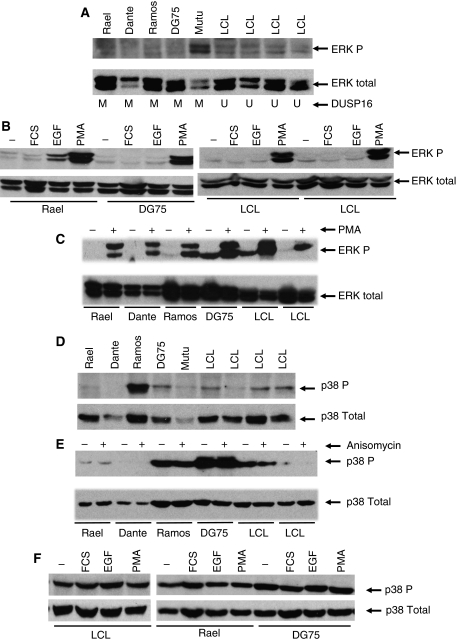
ERK and p38 activity is independent of the methylation status and expression of DUSP16 in BL. (**A**) Constitutive ERK activity is not elevated in BL relative to LCLs and is unrelated to the expression and methylation status of DUSP16. Protein lysates were prepared from exponentially growing BL cell lines and LCL as indicated and ERK activity determined by western blotting as described in Materials and Methods section. (**B**) ERK phosphorylation is induced by PMA but not serum or EGF in BL and LCL. The indicated BL cell lines were serum-starved overnight then challenged with 40% serum (FCS), EGF or phorbol ester (PMA) as indicated and both total ERK and phosphorylated ERK levels were determined by western blotting as described in Materials and Methods section. Only phorbol ester reproducibly and robustly induces ERK activity under these conditions. (**C**) Inducible ERK phosphorylation is not influenced by the methylation status of DUSP16. The indicated cell lines were serum-starved overnight then treated with PMA as indicated and both total ERK and phosphorylated ERK determined by western blotting as described in Materials and Methods section. (**D**) Constitutive p38 activity is not elevated in BL relative to LCL and is unrelated to the expression and methylation status of DUSP16. Protein lysates were prepared from exponentially growing BL cell lines and LCLs as indicated and total and phosphorylated p38 activity was determined by western blotting as described in Methods. (**E**) Anisomycin does not activate p38 in BL or LCLs. The indicated cell lines were exposed to anisomycin and total and phosphorylated p38 levels were determined by western blotting as described in Materials and Methods section. (**F**) Activation of p38 by mitogens is not related to expression or methylation of DUSP16. The indicated BL cell lines and LCLs were serum-starved overnight then challenged with 40% serum (FCS), EGF or phorbol ester (PMA) as indicated and both total p38 and phosphorylated p38 levels were determined by western blotting as described in Materials and Methods section.

**Figure 4:**
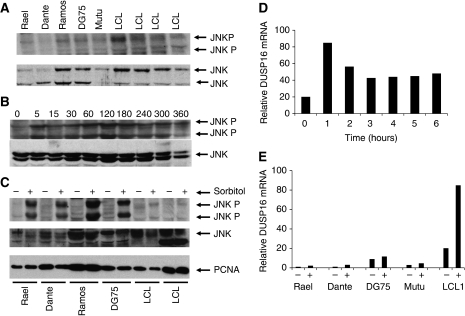
Methylation in the DUSP16 CpG island modulates inducible but not constitutive activation of JNK via inhibition of negative feedback transcriptional upregulation of DUSP16 expression. (**A**) Constitutive JNK expression and activity is not elevated in BL relative to LCL and is unrelated to the expression and methylation status of DUSP16. Protein lysates were prepared from exponentially growing BL cell lines and LCL as indicated and JNK expression and activity were determined by western blotting as described in Materials and Methods section. (**B**) JNK phosphorylation is induced by sorbitol. Ramos BL cells were serum-starved over night, then treated with 0.4 M sorbitol. Cells were collected at the indicated times (min) and total and phosphorylated JNK determined by western blotting as described in Materials and Methods section. JNK phosphorylation is maximal at 120 min after the addition of sorbitol. (**C**) Sorbitol induces JNK activation in BL but not LCL. The indicated BL cell lines were serum starved overnight then challenged with sorbitol. After 30 min, JNK phosphorylation was determined by western blotting as described in Materials and Methods section. (**D**) Sorbitol induces transcriptional upregulation of DUSP16. EBV-immortalised lymphoblastoid cells were exposed to 0.4 M sorbitol and *DUSP16* mRNA levels determined by qPCR at the indicated times (h) as described in Materials and Methods section. Induced levels of *DUSP16* mRNA are maximal at 1 h after addition of sorbitol. Data shown are from a representative experiment. (**E**) Methylation blocks sorbitol-induced upregulation of *DUSP16* mRNA. LCL and BL cell lines, as indicated, were serum-starved overnight, then challenged with 0.4 M sorbitol (+) or not exposed to sorbitol (−). *DUSP16* mRNA levels were determined by qPCR after 1 h as described in Materials and Methods section. Data shown are from a representative experiment.

Next, we tested whether cell lines with methylated DUSP16 exhibit increased MAPK activity. In initial studies, we used immunoblotting to compare the constitutive activity of ERK, p38 and JNK in cycling BL cell lines and LCLs. Although there were variations between individual BL cell lines, constitutive levels of the MAPK were not strikingly elevated in BL cell lines relative to LCL, nor were they related to DUSP16 methylation status (Figures 3A, D and 4A). We next tested whether DUSP16 methylation affected dynamic activation of each of the MAPK, rather than constitutive levels. We first sought to identify agents that selectively upregulate each of the MAPK pathways in B lymphocytes. The BL cell lines and LCLs were serum-starved, then exposed to individual agents and MAPK activity assessed. The phosphorylation of ERK was in general not affected by serum or epidermal growth factor, but there was a strong and consistent activation of ERK by the phorbol ester phorbol 12-myristate 13-acetate (Figure 3B). However, there was no detectable difference in phorbol 12-myristate 13-acetate-induced phosphorylation of ERK between BL cells in which DUSP16 is completely methylated (Rael, Dante and Ramos), partially methylated (DG75) or LCL in which DUSP16 is unmethylated (Figure 3C). For example, in DG75 cells that express modest amounts of DUSP16, activation of ERK occurs to a comparable degree as in Rael (undetectable DUSP16) and LCL (high-level expression of DUSP16).

In the case of p38, there was modest elevation of constitutive phosphorylated p38 levels in Ramos, but in other lines with methylation-dependent silencing of DUSP16, such as Rael and Dante, levels of phosphorylated p38 were not elevated relative to LCL (Figure 3D). We tested whether anisomycin increased the levels of phosphorylated p38, but there was no change in any of the cell lines (Figure 3E). We then tested whether mitogens (serum, epidermal growth factor and phorbol ester) increased p38 phosphorylation in LCL and BL cell lines, but there was no striking increase in any of the other BL lines or LCL after exposure to mitogens (Figure 3F).

Finally, we analysed JNK activity. To establish a time course of JNK activation, we analysed Ramos BL cells that express only very low levels of *DUSP16* mRNA. Cells were serum-starved, exposed to sorbitol and analysed for phosphorylated JNK using western blotting. Phosphorylated JNK was detectable at 5 min and reached maximal levels at 60–120 min (Figure 4B). We then examined the phosphorylation of JNK in the panel of BL cell lines and LCL treated with sorbitol. Each of the BL cell lines with methylation in the DUSP16 CpG island exhibited a strong increase in JNK activation when challenged with sorbitol, whereas there was only a negligible increase in JNK activity in LCL (in which there is no methylation in the DUSP16 CpG island (Figure 4C). These studies thus imply a mechanistic association between methylation in the DUSP16 CpG island and deregulated JNK signalling in response to sorbitol.

### Methylation blocks negative feedback induction of DUSP16 expression

The activation of MAPK signalling under normal physiological conditions results in the transcriptional upregulation of specific *DUSP* gene expression that mediates a negative feedback loop and terminates MAPK activity. We therefore asked whether methylation in the CpG island blocks the negative feedback transcriptional upregulation of DUSP16 after activation of JNK signalling. We initially performed time course analysis of transcriptional induction of DUSP16 in two independent LCLs (in which there is no detectable methylation in the DUSP16 CpG island) treated with sorbitol. There was upregulation of *DUSP16* mRNA in both of the LCLs analysed, with maximum levels attained by 1 h after sorbitol exposure (see representative experiment, Figure 4D). We then tested the induction of *DUSP16* mRNA in the BL cell line panel in comparison to LCL. As previously observed, sorbitol caused a rapid, approximately five-fold induction of *DUSP* mRNA in LCL1. In the Rael and Dante BL cell lines, with dense methylation in the DUSP16 CpG island, induction of *DUSP16* mRNA was greatly reduced in both cell lines relative to that seen in LCL (Figure 4E). In Mutu and DG75, in which there is partial methylation in the DUSP16 CpG island, there was a slightly stronger induction of *DUSP16* mRNA, but this too was reduced in magnitude relative to the LCL (Figure 4E). These results reveal, therefore, that methylation in the DUSP16 CpG island not only blocks constitutive expression of DUSP16, but also inhibits the normal, physiological transcriptional upregulation of DUSP16, which occurs in response to stress such as that imposed by sorbitol.

**Figure 5:**
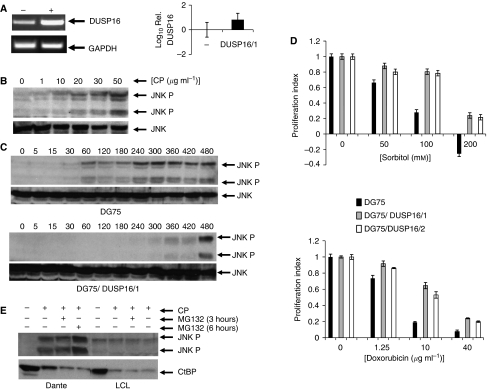
DUSP16 expression modulates JNK activation and chemosensitivity in BL. (**A**) Ectopic expression of *DUSP16* mRNA in DG75 cells transfected with DUSP16 expression vectors. DG75 cells were stably transfected with a DUSP16 expression plasmid as described in Materials and Methods section. *DUSP16* mRNA levels were determined by RT–PCR (left panel) and qPCR (right panel) as described in Materials and Methods section. RT–PCR shows parental (−) and DUSP16-transfected (+) cell line analysed for DUSP16 and the control gene *GAPDH*. The qPCR shows the log_10_ of *DUSP16* mRNA levels in one such clone of transfected cells (DUSP16/1) relative to parental DG75 cells (−)±1 s.d. (triplicate analyses). (**B**) Cisplatin induces dose-dependent activation of JNK in BL cells. Dante cells were exposed for 6 h to the indicated concentrations of cisplatin, after which cells were collected and total and phosphorylated JNK levels determined by western blotting as described in Materials and Methods section. JNK phosphorylation increases with increasing cisplatin concentration. (**C**) Ectopic expression of DUSP16 inhibits drug-induced activation of JNK. Parental DG75 cells and the clone DG75 DUSP16/1 were exposed to cisplatin (50 *μ*g ml^−1^). Cells were collected at the indicated times (min) and the level of total and phosphorylated JNK determined by western blotting. Activation of JNK is detectable at 60 min in the parental cells, but not until 360 min in the cells ectopically expressing DUSP16. (**D**) Ectopic expression of DUSP16 reduces sensitivity of BL cells to cytotoxic drugs. Parental DG75 cells (black columns), together with two independent clones ectopically expressing DUSP16 (denoted DG75/DUSP16/1 (grey columns) and DG75/DUSP16/2 (white columns), were grown to logarithmic phase, then exposed to varying concentrations of sorbitol (mM) and doxorubicin (*μ*g ml^−1^) as indicated. Proliferation was assessed using the CellTiter 96 Aqueous One solution cell proliferation assay (Promega) according to the manufacturer's instructions 48 h after addition of drug. Each point was determined in quadruplicate. Data shown are mean±1 s.d. (**E**) CtBP degradation in drug-treated cells is not influenced by JNK phosphorylation or DUSP16 status. Dante BL (in which DUSP16 is fully methylated) and LCLs were exposed to 50 *μ*g ml^−1^ cisplatin for 6 h with or without MG132 for 3 or 6 h as shown, then subjected to western blotting analysis of phosphorylated JNK and CtBP. JNK phosphorylation occurs only in Dante cells, whereas CtBP degradation is seen in both cell lines and is unaffected by proteasome inhibition.

These data suggest a potential role for DUSP16 in suppressing activation of JNK signalling in response to stress, such as that imposed by sorbitol, and imply that methylation in the DUSP16 CpG island potentiates JNK signalling. As such, it was of obvious interest to determine whether ectopic expression of DUSP16 could re-establish negative feedback of JNK signalling in cells lacking endogenous expression. To address this possibility, we attempted to ectopically express DUSP16 in Rael, Dante and Ramos BL cell lines in which expression of the endogenous gene is undetectable. However, despite multiple, repeated attempts, it was not possible to establish stable derivatives of any of these cell lines. We therefore ectopically expressed DUSP16 in DG75 cells in which there is a modest level of endogenous DUSP16 expression. We isolated several clones in which DUSP16 expression was increased relative to parental DG75 cells (Figure 5A). We wished to test JNK activation by clinically used chemotherapeutic agents. We therefore investigated whether cisplatin induced JNK in Dante BL cells and observed a dose-dependent increase in JNK phosphorylation, confirming that cisplatin activates JNK in BL (Figure 5B). Next, we compared parental DG75 cells with daughter clones ectopically expressing DUSP16. JNK was robustly activated by cisplatin with phosphorylated JNK detectable within 30 min of exposure to cisplatin. In contrast, phosphorylation of JNK was suppressed in the DUSP16 ectopically expressing cell line, phosphorylated JNK only becoming detectable at 300 min (Figure 5C).

### DUSP16 regulates cellular sensitivity to cytotoxic drugs in BL

Differences in JNK activation prompted us to test, in proliferation assays, the sensitivity to cytotoxic agents of cell lines expressing different levels of DUSP16. Sorbitol and doxorubicin demonstrated clear, dose-dependent cytotoxicity to parental DG75 cells (Figure 5D). Two independently derived clones of DG75 cells ectopically expressing DUSP16 were reproducibly less sensitive to sorbitol and doxorubicin than parental DG75 cells, showing a two to four-fold increased resistance factor (Figure 5D). A similar effect of DUSP16 expression on sensitivity to cisplatin was also seen (data not shown). In some systems, JNK activation promotes ubiquitin-dependent proteolysis of the transcriptional co-repressor CtBP (Wang *et al*, 2006). If a similar mechanism operates in BL, we would predict that the strongly enhanced activation of JNK seen in cells with methylated DUSP16 would cause increased degradation of CtBP. To test this possibility, we asked whether levels of CtBP varied with the status of DUSP16 methylation and hence inducibility of JNK activity. Dante BL cells (complete methylation of DUSP16) and LCLs were treated for 6 h with cisplatin in the presence or absence of the proteasome inhibitor MG132 and levels of phosphorylated JNK and CtBP analysed. Exactly as seen previously in Ramos and DG75 cells, cisplatin induced phosphorylation of JNK, but there was no increase in JNK phosphorylation in the LCL cells (Figure 5E). However, in both cell lines, levels of CtBP decreased irrespective of DUSP16 status and JNK phosphorylation. Furthermore, the decrease in CtBP levels was not inhibited by MG132 (Figure 5E). Together, these results imply that the enhanced activation of JNK seen in DUSP16-deficient cells is not associated with enhanced proteolysis of CtBP.

## Discussion

In this study, we show that the type IV DUSP, DUSP16, is subject to methylation-dependent transcriptional silencing in BL, resulting in the deregulation of JNK signalling. There are a large number of *DUSP* genes in the human genome, but relatively little is known regarding their involvement in cancer. The type I DUSP, DUSP1, has been identified previously in studies using 5′AZA to pharmacologically reverse methylation, yet the CpG island was reported to be unmethylated (Rauhala *et al*, 2005). Furthermore, DUSP6 has been reported to be a target for methylation-dependent silencing in pancreatic cancer (Xu *et al*, 2005). A single previous study in prostate cancer has reported transcriptional downregulation of DUSP16 expression, but the mechanistic basis of downregulation was not established (Kibel *et al*, 2004). The data we present are, therefore, to the best of our knowledge, the first demonstration of epigenetic silencing of a type IV *DUSP* gene in human cancer. The ectopic expression of DUSP16 in fibroblasts transformed by BCR-ABL reduces their transforming activity *in vivo* accompanied by downregulation of BCR-ABL-induced activation of JNK (Hoornaert *et al*, 2003). These studies support the candidacy of DUSP16 as a tumour suppressor and are consistent with our observations of a role for DUSP16 in modulating the intensity and duration of JNK signalling.

Although DUSP16 is commonly methylated in BL cell lines and endemic, sporadic and human immunodeficiency virus-associated BL, methylation was almost never detected in EBV-immortalised LCLs and the gene was abundantly expressed in these cells, implying that methylation is specific to neoplasia. Moreover, we did not detect methylation of *DUSP16* in any cancer except BL, including analysis of a panel of NHL cell lines and clinical cases of DLBCL, FL, MCL and marginal zone lymphoma. As such, these results suggest a clear specificity of DUSP16 methylation for BL. Furthermore, analysis of the breast and ovarian cancer cell lines and malignant melanoma cell lines also failed to detect methylation. Why is methylation-dependent silencing of DUSP16 so tightly restricted to BL and not observed in multiple other B lymphoma subtypes? DUSP16 is a direct transcriptional target of B lymphocyte-induced maturation protein, Blimp-1 (Magnusdottir *et al*, 2007), suggesting that it may have a role in the normal developmental physiology of B lymphocytes. It remains to be determined, however, whether DUSP16 is downregulated in other cancers by genetic or other mechanisms. In this respect, it is noteworthy that the *DUSP16* gene is located on chromosome 12p, an area of frequent chromosomal loss in human tumours. In leukemia, recent data suggests that deletions of 12p are a final event in mature cells (Wiemels *et al*, 2008). In non-small cell lung cancer, 12p deletions are an early occurrence (Grepmeier *et al*, 2005).

We previously reported that the Polo-like kinase *Snk/Plk2* was subject to methylation-dependent transcriptional silencing at high frequency in BL (Syed *et al*, 2006). Methylation of *Snk/Plk2* was, however, also detected in multiple other B cell neoplasias, whereas methylation in DUSP16 seems to be tightly restricted to BL. We have sought to address the mechanistic basis favouring the selection of clones lacking DUSP16. Our results reveal that methylation of *DUSP16* correlates closely with deregulation of JNK signalling. The activation of JNK signalling has been reported to promote either cell death (Wang *et al*, 2006) or cell survival according to different studies, although the reasons for such differences in effect are not known. The data we present show that JNK activation, at least in the context of BL, promotes apoptosis and cytotoxicity in cells challenged with cytotoxic and/or oxidative stress, as ectopic expression of DUSP16 reduced activation of JNK and this correlated with reduced sensitivity to doxorubicin, sorbitol and cisplatin. One implication of the association of JNK activation with cell death is that loss of JNK activation may represent a mechanism of clinical drug resistance. Evidence in acute myeloid leukemia cell lines to support this possibility has recently been published (Lagadinou *et al*, 2008). It would be of obvious interest to analyse the expression and epigenetic status of DUSP16 in relapsed BL (at which time there are few active chemotherapeutic options) to determine whether changes in methylation in the DUSP16 CpG island occur with acquisition of clinical drug resistance.

Promotion of apoptosis by activation of JNK has been shown in some systems to result from enhanced degradation of the transcriptional co-repressor CtBP (Wang *et al*, 2006). We tested whether degradation of CtBP was increased in BL with DUSP16 methylation, but we observed no evidence to support this hypothesis, CtBP degradation being similar irrespective of DUSP16 status and unaffected by treatment with the proteasome inhibitor MG132.

In conclusion, we have identified *DUSP16* as a new epigenetically regulated gene inactivation of which seems specific to BL. We present evidence that expression of DUSP16 expression selectively modulates the activation of JNK and influences cellular sensitivity to chemotherapeutic agents.

## Figures and Tables

**Table 1:** Methylation of the DUSP16 CpG island in B cell neoplasia

**Cell line**	**Origin**	**EBV**	**DUSP16 methylation (MSP)**
AW Ramos	Endemic	+	+/−
BL2	Sporadic	−	−
BL41	Sporadic	−	+
Dante	Sporadic	+	+
DG75	Sporadic	−	+/−
Mak-I	Not known	+	+/−
Mutu	Endemic	+	+/−
Rael	Sporadic	+	+
Ramos	Endemic	−	+/−
			
**Tissue**	**Origin**	**DUSP16 methylation (MSP)**	
DLBCL		0/10	
FL		0/10	
MCL		0/10	
MZL		0/10	
BL	Endemic	27/45	
BL	Sporadic	2/8	
BL	HIV-associated	2/4	

Abbreviations: BL=Burkitt's lymphoma; DLBCS=diffuse large cell lymphoma; DUSP16=dual specificity phophatase-16; EBV=Epstein–Barr Virus; FL=follicular lymphoma; HIV=human immunodeficiency virus; MCL=mantle cell lymphoma; MSP=methylation-specific PCR; MZL=marginal zone lymphoma.
